# Supplementary material for: Zinc-finger BED domains drive the formation of the active Hermes transpososome by asymmetric DNA binding
Source: Nat Commun. 2023 Jul 25;14:4470. doi: 10.1038/s41467-023-40210-3 (PMC10368747; doi:10.1038/s41467-023-40210-3)
Supplement: Supplementary file 1 — Supplementary Information [file 41467_2023_40210_MOESM1_ESM.pdf]

Supplementary Information

Zinc-finger BED domains drive the formation of the active *Hermes* transpososome by asymmetric DNA binding

Laurie Lannes[1], Christopher M. Furman[1], Alison B. Hickman[1] and Fred Dyda[1]*

[1]Laboratory of Molecular Biology, National Institute of Diabetes and Digestive and Kidney Diseases, National Institutes of Health, Bethesda, MD, 20892 USA

*Correspondence: fred.dyda@nih.gov

This document includes Supplementary Figures 1-12, Supplementary Tables 1-4

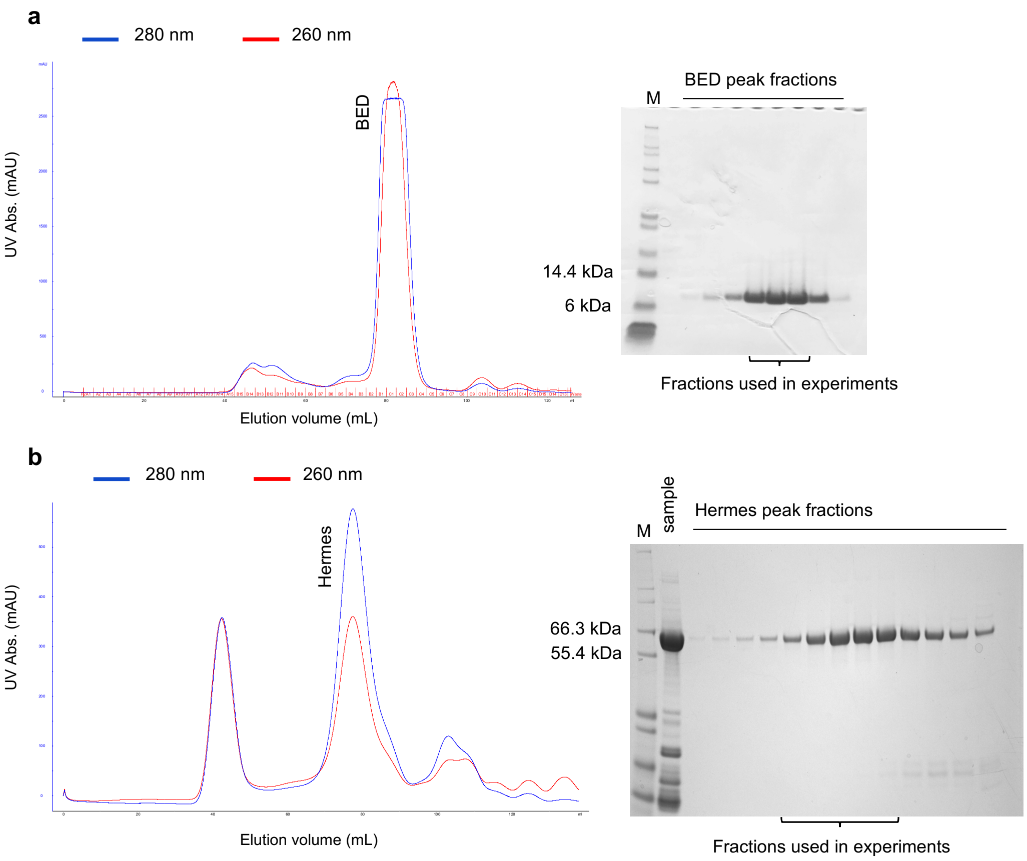

Figure 1. Final purification step (size exclusion chromatography or SEC) of the Hermes BED domain (**a**) and of the full-length Hermes transposase (**b**). The SEC chromatograms are on the left and the SDS-PAGE analysis of the collected fractions are on the right. M: protein size marker (Mark12, Novex).

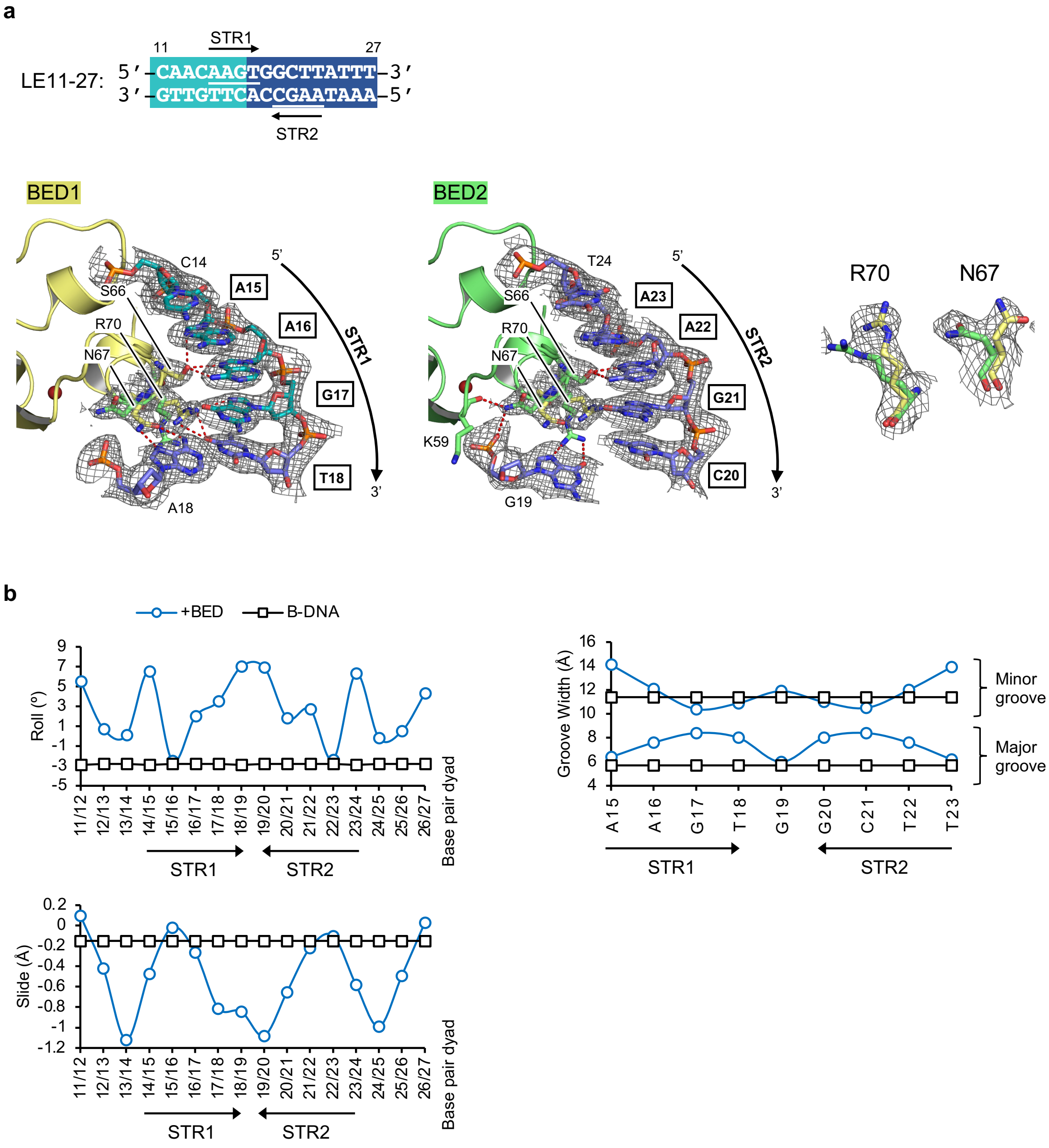

Figure 2. **Details of the LE11-27/BED crystal structure. a** The hydrogen-bonding network (red dashed lines) between the residues S66, N67 and R70 of the Hermes BED domain with the LE-STR1 and LE-STR2 of the LE11-27 DNA. The residues N67 and R70 adopt a different conformation when interacting either with the STR1 (yellow) or the STR2 (green). The electron density (2fofc) is displayed as a grey mesh at sigma 0.7. **b** The roll, slide and groove parameters of the LE11-27 DNA bound to Hermes BED domains (crystal structure from Fig. 2, PDB: 8EB5) compared to that of measured in its B-form.

a First Assessment

Hermes transposon left-end (17 STR)

```
    |5        |15       |25       |35       |45       |55       |65
CAGAGAACAACAACAAGTGGCTTATTTTGATACTTATGCGCCACTTGCTACTTATGAGTACAATTGT
GCTTTGCCACTTGAACAAAAAATTCATTGATTCATCGACACTCGGGTATGTTTTGTCGTGTCGTTCT
GCGCACTCAGTTAAATTTTTTGTCTTACTCTCTTGCTCTCAGCACATCAAGTGTTGTTACTTGTTGT
TACTCAGTCGCCTGCCTTATGCTTTTTGGAGAGCGAAAGCACAACGATCAGAACGGAGAAGTAACAA
CTTGTTTTGCTAACAAGTGGCTTATGCACTTGAGTGTGTTTTACACATGTTTTTGAGTTTCGCAGCA
AAATGTTCCGATTTGAGCACAATAATTTTACCGTTATTTTGAGTTTTTTAGTTTTGAATAATAAATG
TGATTTACTGTTCATCCTCAAAAGAGTTTAAGCAGTAGTAGAGATTAG
```

Hermes transposon right-end (14 STR)

```
    |5        |15       |25       |35       |45       |55       |65
CAGAGAACTTCAACAAGCCACAGGCAAACGTAAGCCACATAGATAAGCACAAGTGTTTTGGGTGTCA
AGCCTTTTGAGTGCAAGTATTTTTTTATACACGAGTATTTTTTCACAACTTAACAACAACAGTTGTT
TGATGTTAAGATCACCGCTAGAGTATGAGAGAGTAAAAAGTGTACAAACTCACAAGTGGACGTGTGC
GATTTGTCAATTGGCAAATTATACTCACTTCTTGTTGTGTTGTATACTCATTGAACATGAGGGTTGT
GTGTGTGTGCTATTGTTTGTAGTATTGCATATGATTCTGTACAAGACTAGGAAAAGAGCATAAGTAT
ATTAAAAAAAAAAGAAAAATGCTTCTTTTAAACTTAAATTTTTCCAGTCCAAAATTTATTTATTTTT
TTTTTTTATTTTAACAAACAAACTTATAAAAAATATTCTTTTTATTTAAAAATGTGTAA
```

b Second Assessment

Hermes transposon left-end (11 STR and putative BED binding sites)

```
    |5        |15       |25       |35       |45       |55       |65
CAGAGAACAACAACAAGTGGCTTATTTTGATACTTATGCGCCACTTGCTACTTATGAGTACAATTGT
GCTTTGCCACTTGAACAAAAAATTCATTGATTCATCGACACTCGGGTATGTTTTGTCGTGTCGTTCT
GCGCACTCAGTTAAATTTTTTGTCTTACTCTCTTGCTCTCAGCACATCAAGTGTTGTTACTTGTTGT
TACTCAGTCGCCTGCCTTATGCTTTTTGGAGAGCGAAAGCACAACGATCAGAACGGAGAAGTAACAA
CTTGTTTTGCTAACAAGTGGCTTATGCACTTGAGTGTGTTTTACACATGTTTTTGAGTTTCGCAGCA
AAATGTTCCGATTTGAGCACAATAATTTTACCGTTATTTTGAGTTTTTTAGTTTTGAATAATAAATG
TGATTTACTGTTCATCCTCAAAAGAGTTTAAGCAGTAGTAGAGATTAG
```

Hermes transposon right-end (8 STR and putative BED binding sites)

```
    |5        |15       |25       |35       |45       |55       |65
CAGAGAACTTCAACAAGCCACAGGCAAACGTAAGCCACATAGATAAGCACAAGTGTTTTGGGTGTCA
AGCCTTTTGAGTGCAAGTATTTTTTTATACACGAGTATTTTTTCACAACTTAACAACAACAGTTGTT
TGATGTTAAGATCACCGCTAGAGTATGAGAGAGTAAAAAGTGTACAAACTCACAAGTGGACGTGTGC
GATTTGTCAATTGGCAAATTATACTCACTTCTTGTTGTGTTGTATACTCATTGAACATGAGGGTTGT
GTGTGTGTGCTATTGTTTGTAGTATTGCATATGATTCTGTACAAGACTAGGAAAAGAGCATAAGTAT
ATTAAAAAAAAAAGAAAAATGCTTCTTTTAAACTTAAATTTTTCCAGTCCAAAATTTATTTATTTTT
TTTTTTTATTTTAACAAACAAACTTATAAAAAATATTCTTTTTATTTAAAAATGTAA
```

Figure 3. **Mapping on the *Hermes* transposon ends of the subterminal repeats (STR) proposed as putative BED binding sites. a** Mapping based on the definition of the STR as 5'-AAGY-3' with Y being T or C. **b** Mapping based on a finer definition of the BED binding site that corresponds to 5'-AAGT-3' followed by an AT-rich sequence or in the special situation of the quasi-palindrome 5'-AAGTGGCTT-3'. The LE-STR1-STR2 quasi palindrome interacts with two cooperative BED domains is highlighted by a red box.

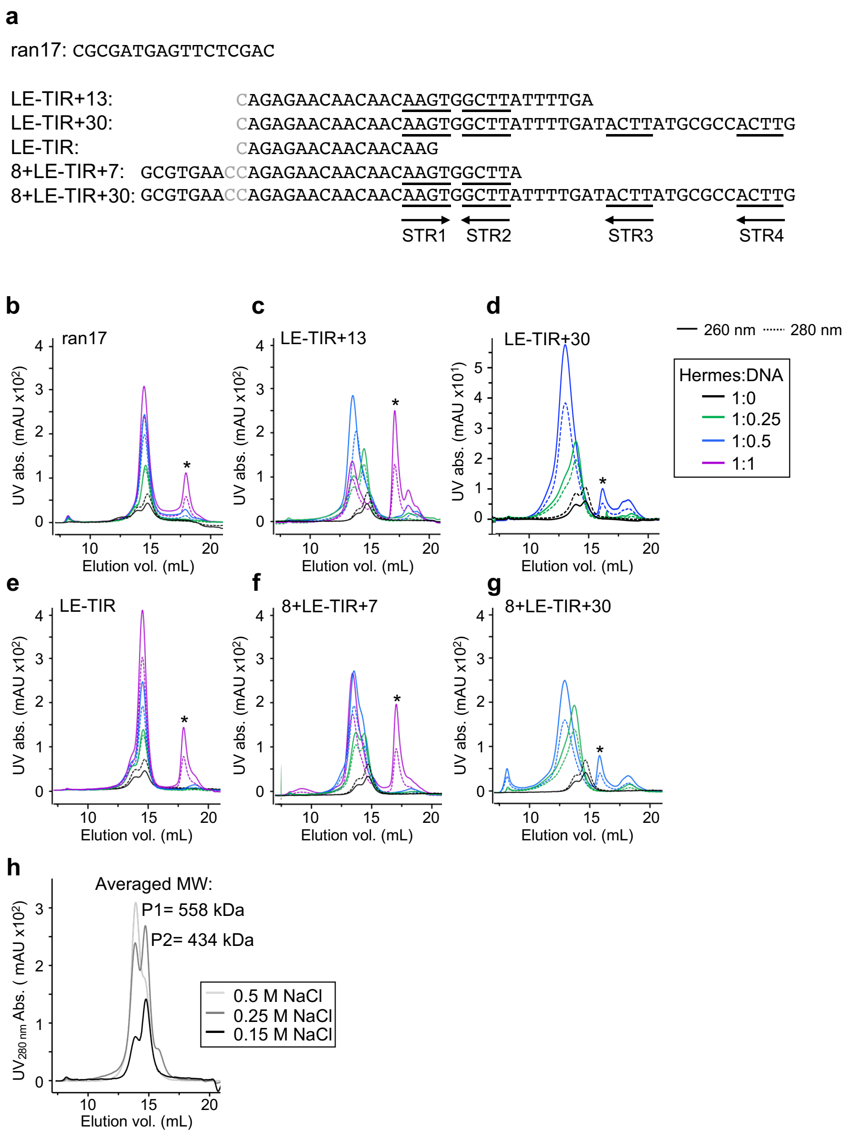

Figure 4. **Analysis of the Hermes transposase in complex with various DNAs mimicking the left-end (LE) in its cleaved or nicked state by size exclusion chromatography (SEC).**
a Sequence of the top strand of the double-stranded DNAs used in b-g. The bases in grey were absent on the top strand, but their complementary bases were present on the bottom strand. The subterminal repeats STR1 to STR4 are underlined and their orientation is indicated by arrows. **b-g** SEC chromatograms of the Hermes/DNA samples (buffer condition: 25 mM HEPES.Na pH 7.5, 150 mM NaCl). The Hermes-to-DNA ratios are shown in the insert on the right. The free DNA elution peaks are marked by a star (*). H) SEC chromatogram of purified Hermes (in 0.75 M NaCl) dialyzed against three different elution buffers containing 0.5 M (light grey), 0.25 M (grey) and 0.15 M (black) NaCl. The apparent molecular weights (MW) derived from standard curve for two Hermes oligomeric populations, P1 and P2, are indicated.

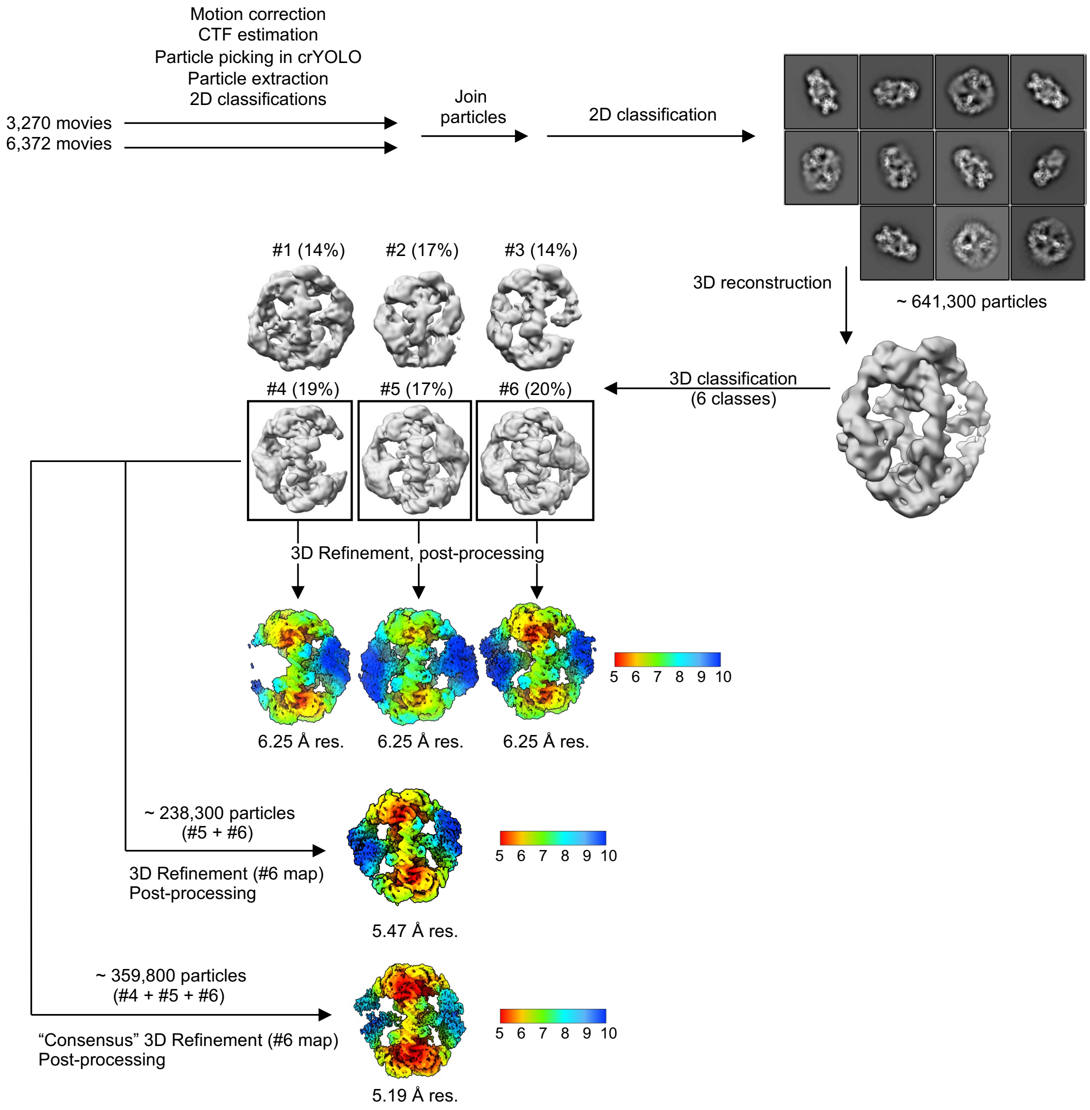

Figure 5. Early stages of the single particle analysis of the cryo-electron microscopy data of the LE-LE transpososome in RELION.

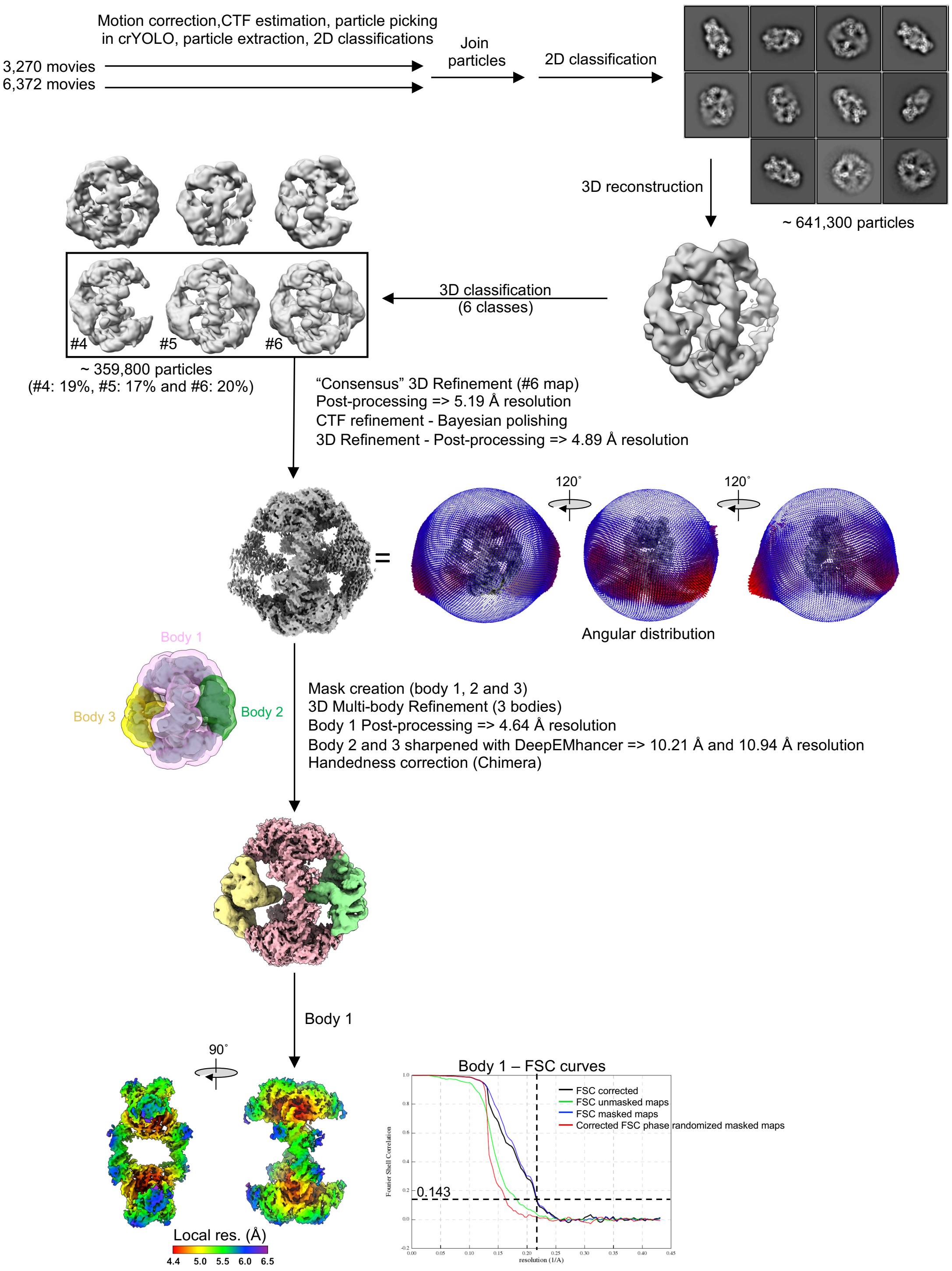

Figure 6. Workflow of the single particle analysis of the cryo-electron microscopy data of the LE-LE *Hermes* transpososome in RELION.

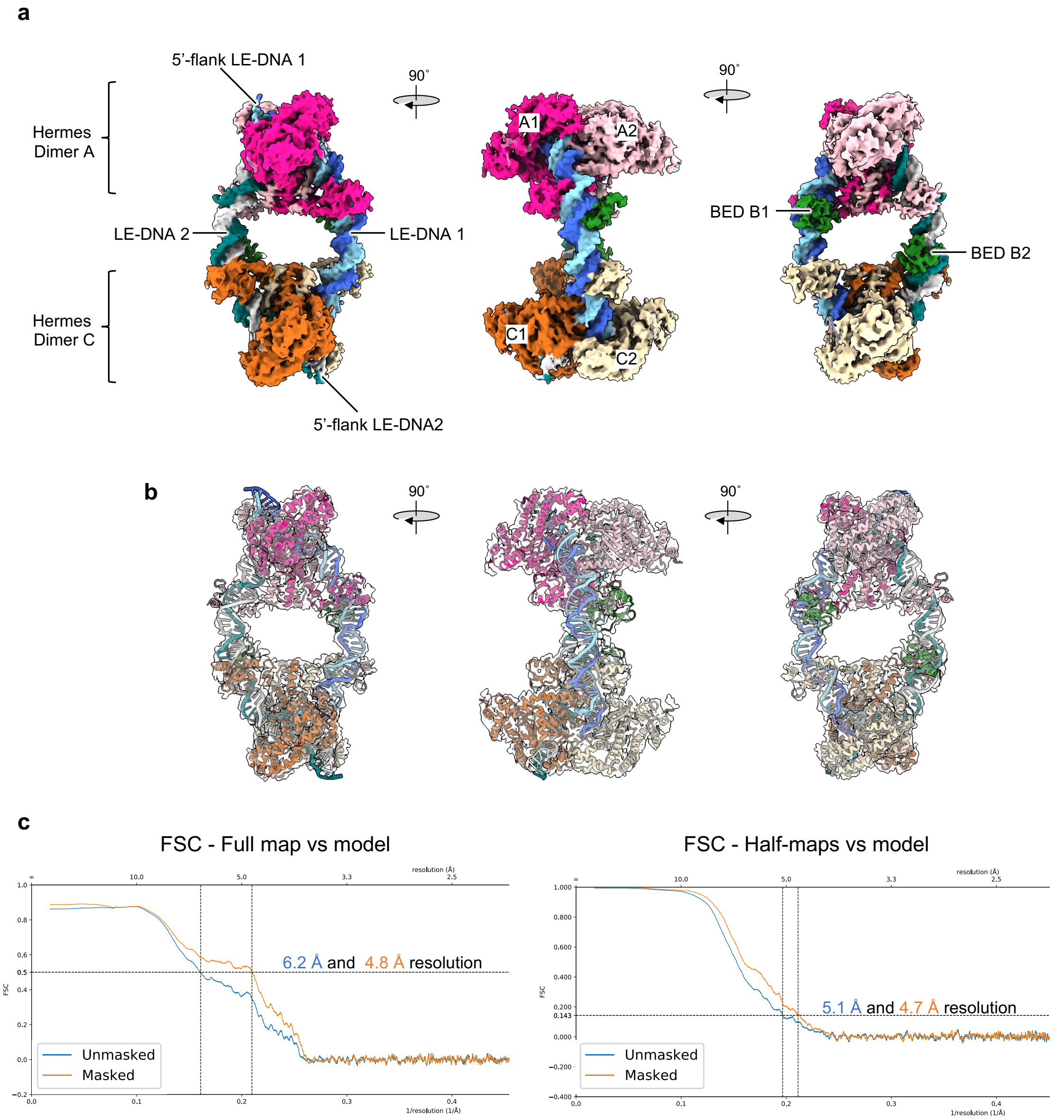

Figure 7. **Model fitting of the core of the LE-LE *Hermes* transpososome inside the Body 1 cryo-EM map of the multi-body refinement. a** RELION final Body 1 map colored to highlight the transposase monomers and DNA strands. **b** Atomic model of the complex build inside the cryo-EM map. The model is color coded as in a. **c** Maps-to-model gold standard FSC curves.

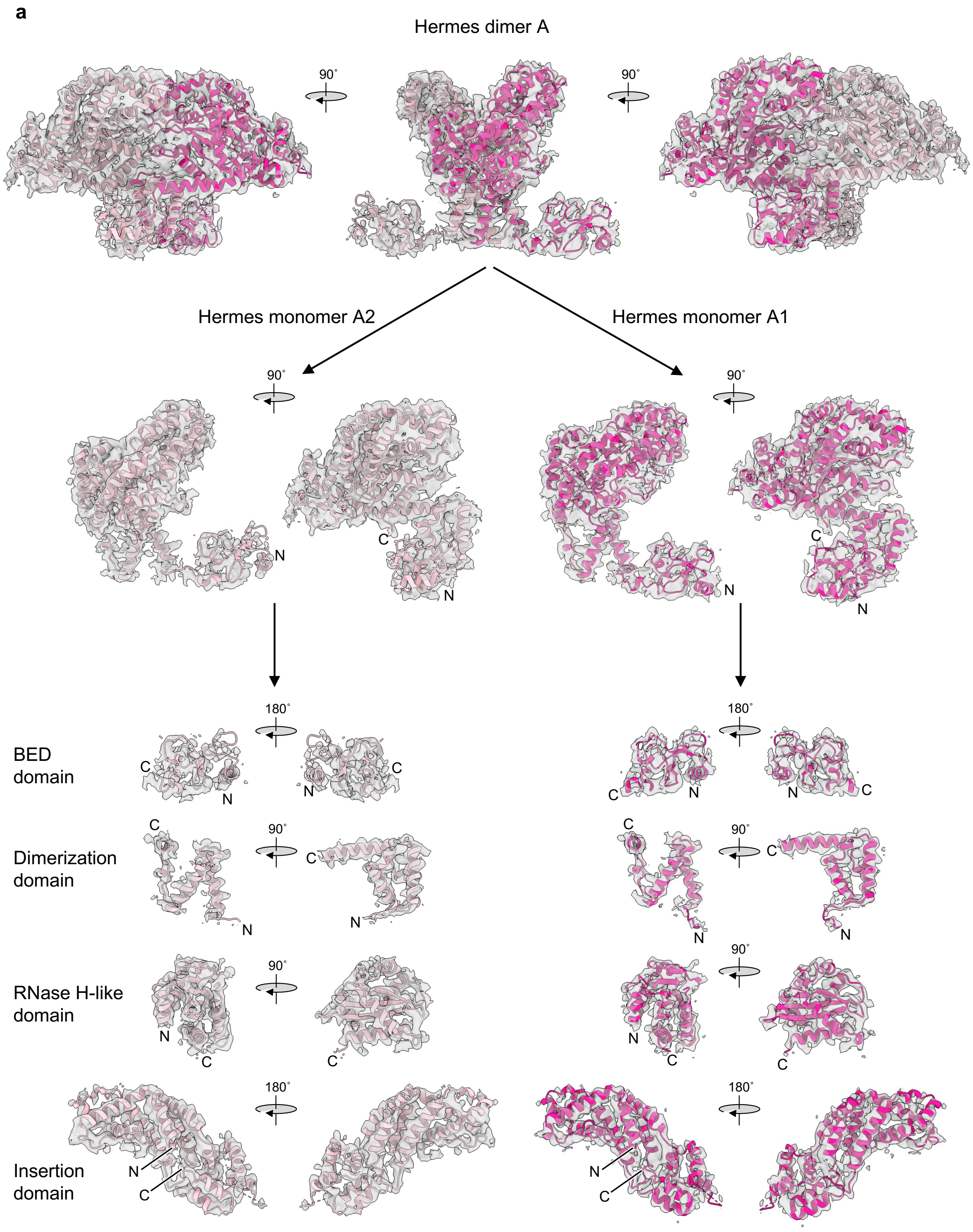

Figure 8. (follows on next two pages)

b

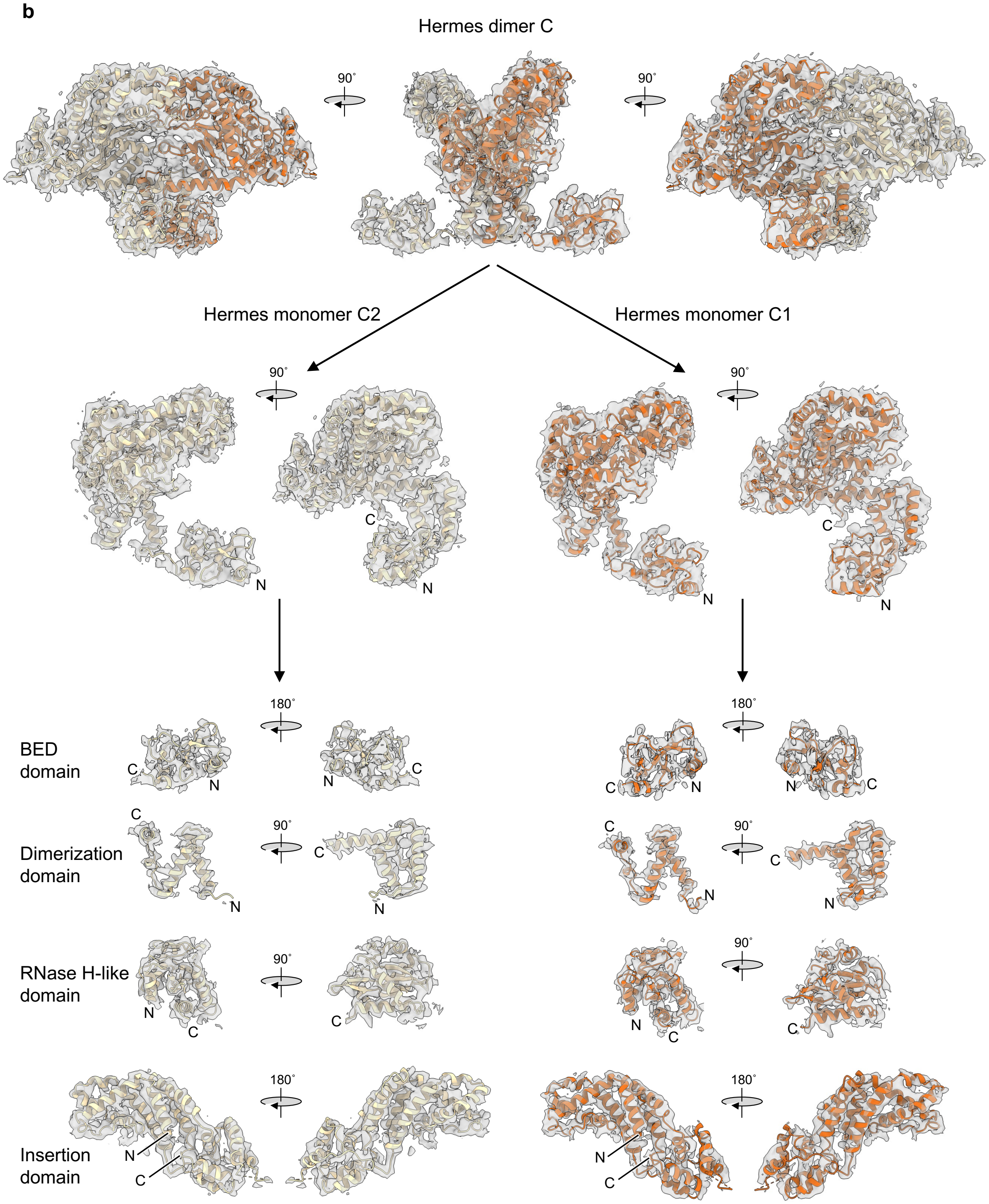

Figure 8. (follows on next page)

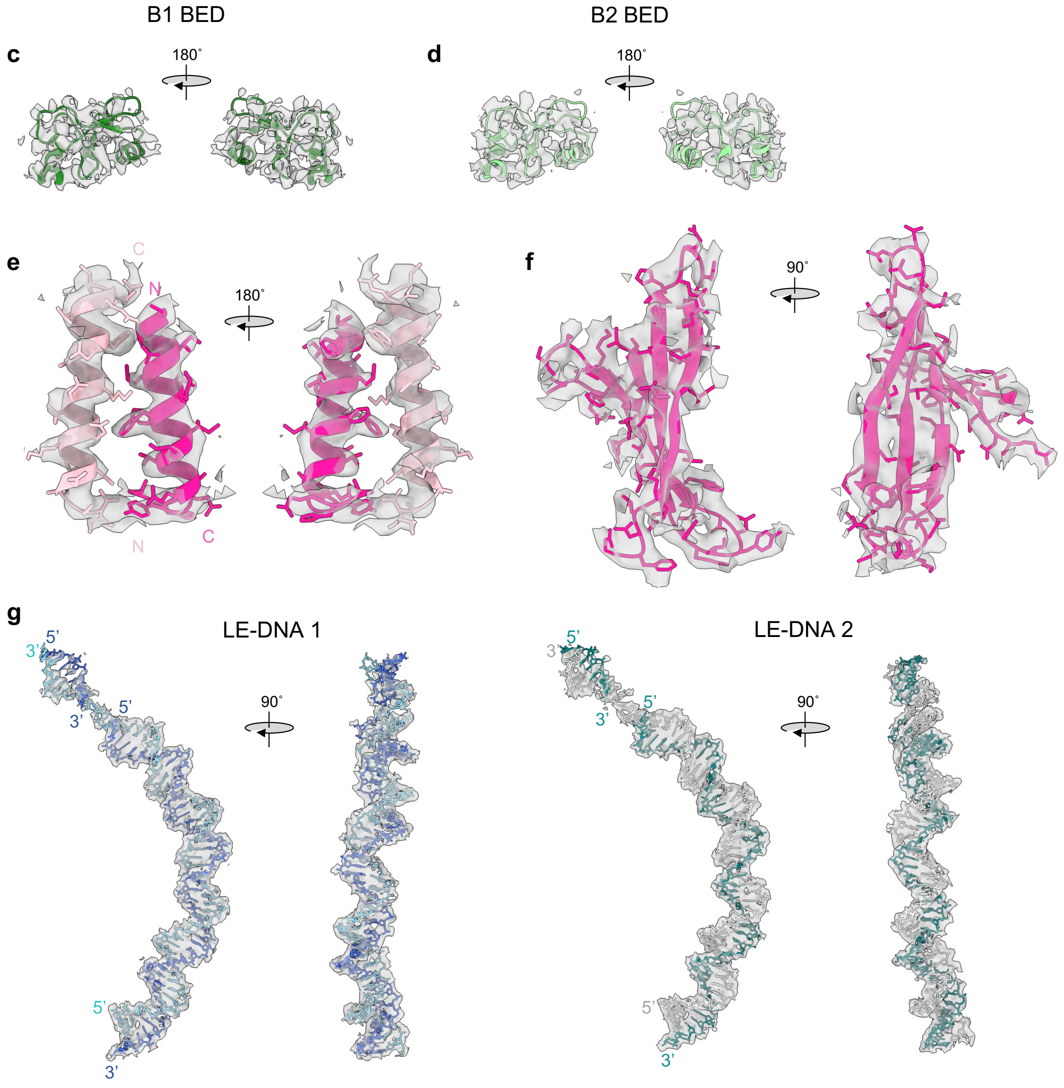

Figure 8. **Regions of the cryo-EM potential map of the core of the LE-LE *Hermes* transpososome (RELION's postprocessed map, Body 1) superimposed with its atomic model. a-b** Dissection of the Hermes dimers A and C. **c** The BED domain of Hermes B1. **d** The BED domain of Hermes B2. **e** α-helix 1 (light pink) and α-helix 2 (dark pink) of the dimerization domain of the dimerized Hermes A2 and A1, respectively. **f** The β-sheet of the RNase H-like domain of Hermes A1. **g** The double-stranded LE-DNAs.

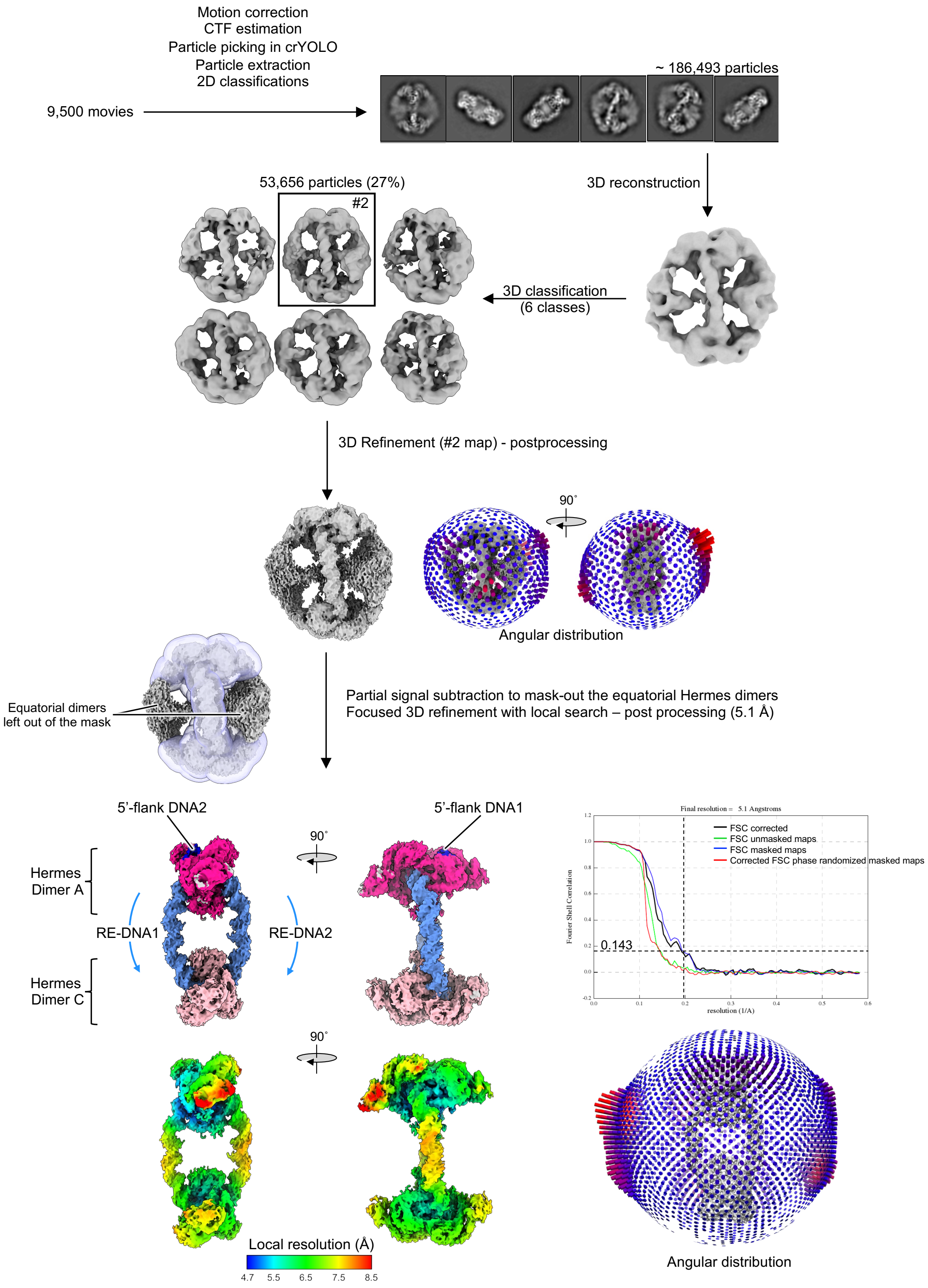

Figure 9. Workflow of the single particle analysis of the cryo-electron microscopy data of the RE-RE *Hermes* transpososome in RELION.

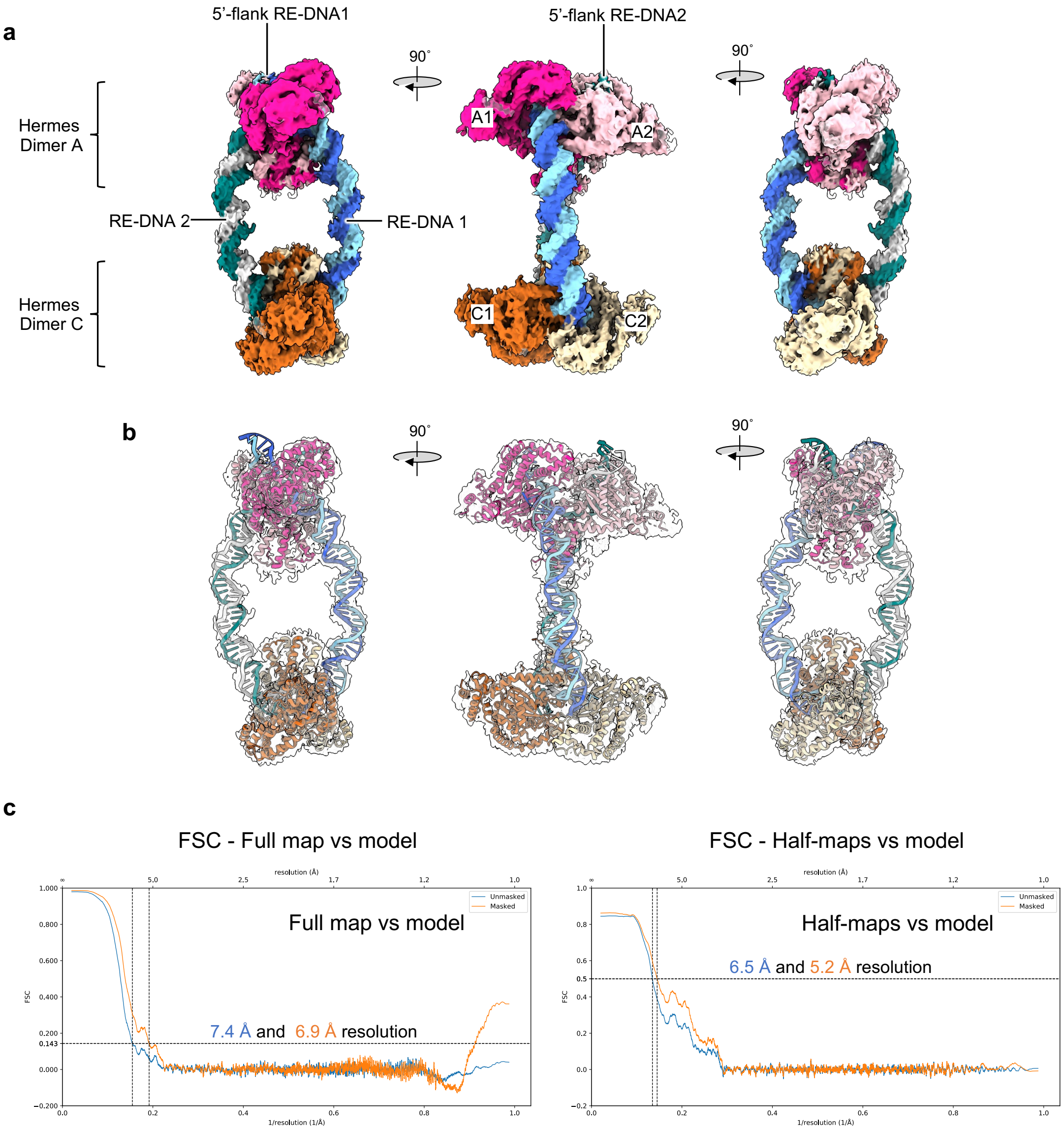

Figure 10. **Model fitting of the core of the RE-RE *Hermes* transpososome inside its cryo-EM map. a** RELION Final map colored to highlight the transposase monomers and DNA strands. **b** Atomic model of the complex build inside the cryo-EM map. The model is color-coded as in a. **c** Maps-to-model gold standard FSC curves.

a

		Binding activity
ran17:	CGCGATGAGTTCTCGAC	+
RE11-27:	CAACAAGCCACAGGCAA	-
RE11-27T:	CAACAAG**T**CACAGGCAA	-
LE11-27:	CAACAAGTGGCTTATTT	+++
LE11-27mut:	CAACAAGTG**TT**TTATTT	+++
LE11-27mutC:	CAACAAG**C**G**TT**TTATTT	-
LE11-27mut-5'G:	C**GG**CAAGTG**TT**TTATTT	+++
LE11-27mut-5'3'G:	C**GG**CAAGTGGC**GC**ATTT	+

b

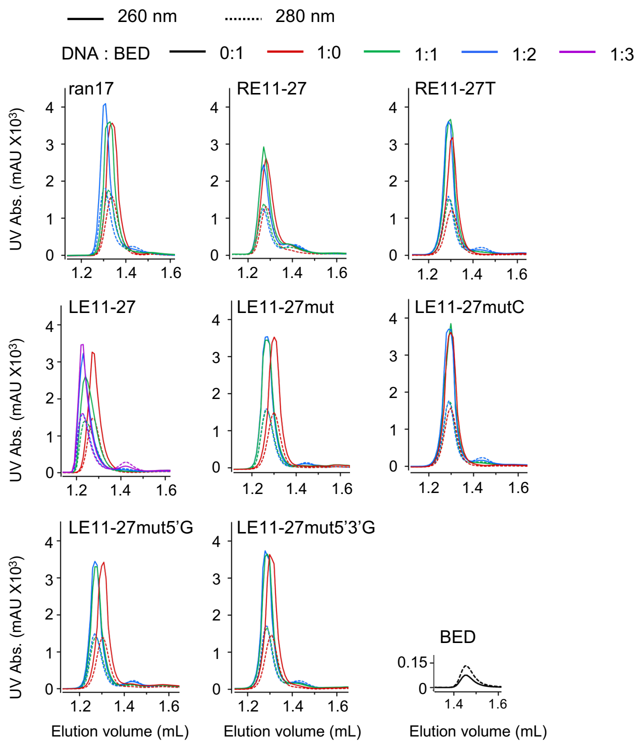

Figure 11. **The minimal BED binding site. a** Top strand of the left-end (LE) and right-end (RE) DNAs used in the interaction assay. The 5'-AAGY-3' (with Y = C or T) motifs are underlined, and the mutations are in bold. The table summarizes the binding activity of the Hermes BED domain for each oligo as determine in b. **b** Size exclusion chromatograms of the BED/DNA mixes. The BED-to-DNA ratios of the samples are reported on top.

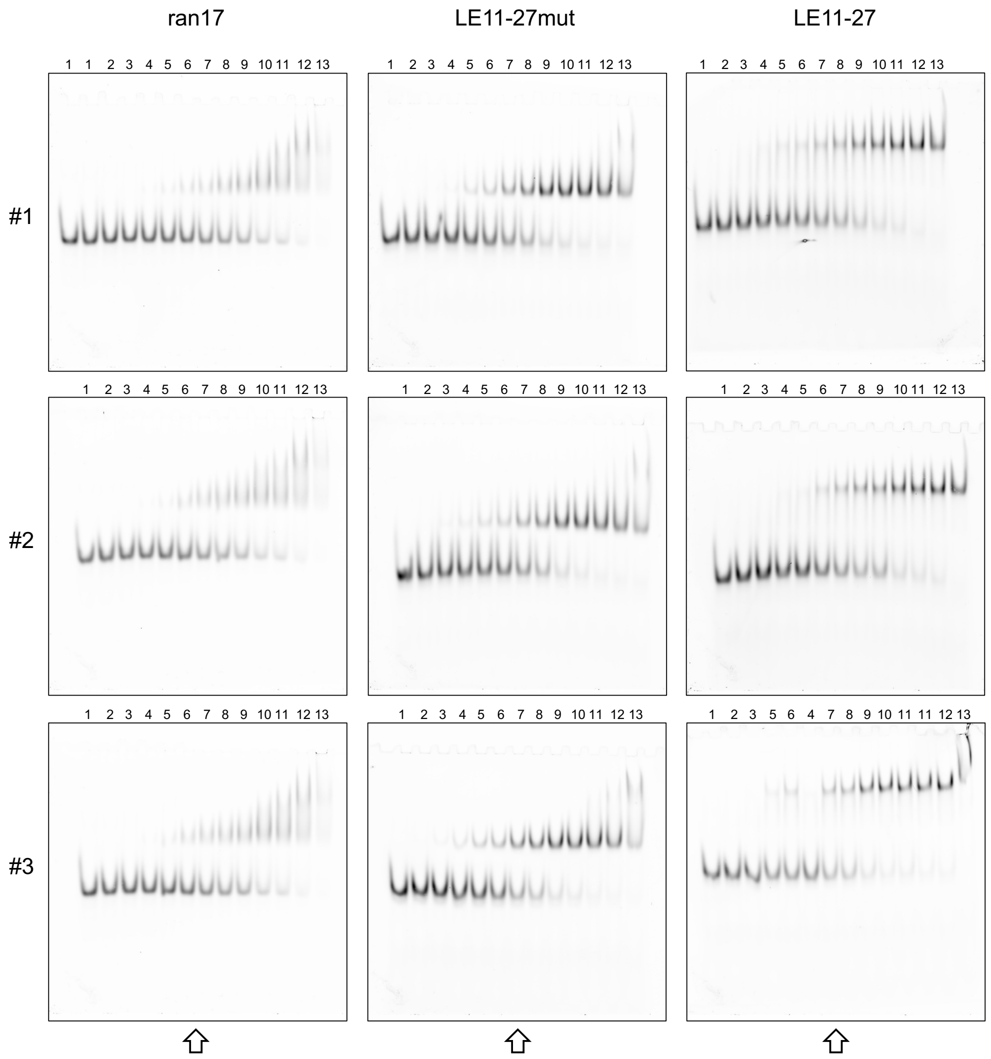

Lane #	[BED] µM
1	0
2	0.25
3	0.5
4	0.75
5	1
6	1.5
7	2
8	2.5
9	3
10	4
11	5
12	10
13	50

Lane #	[BED] µM
1	0
2	0.1
3	0.25
4	0.5
5	0.75
6	1
7	1.5
8	2
9	2.5
10	3
11	4
12	5
13	10

Lane #	[BED] µM
1	0
2	0.25
3	0.5
4	0.75
5	1
6	1.5
7	2
8	2.5
9	3
10	3.5
11	4
12	5
13	10

Figure 12 (related to Main Text Figure 6). Unedited pictured of the triplicate EMSA gels of 6FAM-labelled ran17, LE11-27mut and LE-11-27 DNAs with Hermes' isolated BED domain. The BED concentration in each lane is indicated in tables at the bottom.

Supplementary Table 1. Crystallographic statistics.

	Zn-MAD			Rotating Anode
	"Edge"	"Peak"	"Remote"	
Wavelength (Å)	1.28335	1.28282	1.23984	1.54184
Resolution range (Å)	27.6-2.5 (2.56-2.5)	27.6-3.0	27.6-3.0	27.6-2.7 (2.77-2.7)
Space group	$P6_522$	$P6_522$	$P6_522$	$P6_522$
Unit cell dimensions a, b, c (Å) and α, β, γ (°)	55.3, 55.3, 212.6 90, 90, 120	55.3, 55.3, 212.6 90, 90, 120	55.3, 55.3, 212.6 90, 90, 120	54.9, 54.9 210.3 90, 90, 120
Total reflections	140742 (12541)	80706[a] (8264)	80598[a] (7871)	107528 (9723)
Unique reflections	12541 (923)	4337[a] (420)	4338[a] (420)	9723 (719)
Multiplicity	11.2 (11.2)	18.6 (19.6)	18.6 (18.7)	11.1 (9.5)
Completeness (%)	99.8 (100)	98.74 (100)	98.94 (100)	99.8 (100)
$I/\sigma(I)$	30.7 (1.9)	41.58 (6.57)	42.48 (10.86)	39.45(1.34)
R_{merge} (%)[a]	0.038 (1.36)	0.05492 (0.3711)	0.05491 (0.2373)	0.035 (1.89)
Phasing				
Anomalous resolution (Å)	2.5	3	3	2.7
Number of sites	1	1	1	13
Anomalous (%)phasing power[b]	2.86	2.37	1.96	0.67
Figure of merit acentric	0.46	-	-	-
Refinement				
Number of reflections used[c]	7282 (694)	-	-	-
R_{work} (%)	23 (30)	-	-	-
R_{free} (%)[d]	24 (44)	-	-	-
Number of non-hydrogen atoms in:				
- macromolecule	1331	-	-	-
- ligand	1	-	-	-
- solvent	5	-	-	-
Average B-factor macromolecule (Å^2)	102.9	-	-	-
Average B-factor ligand (Å^2)	98.3	-	-	-
RMSD bond lengths (Å)	0.01	-	-	-
RMSD angles (°)	1.29	-	-	-
Ramachandran favored (%)	98.6	-	-	-
Ramachandran allowed (%)	1.4	-	-	-
Rotamer outliers (%)	0.00	-	-	-
Clashscore	4.27	-	-	-
PDB code	8EB5	-	-	-

Statistics for the highest-resolution shell are shown in parentheses.
[a] $R_{merge} = \Sigma|I_i - \langle I \rangle|/\Sigma I_i$, where I_i is the intensity of measured reflection and $\langle I \rangle$ is the mean intensity of all symmetry-related reflections.
[b] Phasing Power is $\langle [\, | Fh(calc) | \, / \text{ phase-integrated lack of closure }] \rangle$ where Fh(calc) is the calculated structure factor of the reference scatterers (Zn, P, S).
[c] Friedel's law true.
[d] $R_{free} = \Sigma T||F_{calc}| - |F_{obs}||/\Sigma F_{obs}$, where T is a test dataset of about 3–6% of the total unique reflections randomly chosen and set aside prior to refinement.
RMSD stands for root mean square deviation.

Supplementary Table 2. Oligonucleotides used in the study.

Name	Sequence
ran17	5′-(6FAM)CGCGATGAGTTCTCGAC-3′<br>3′- GCGCTACTCAAGAGCTG-5′
LE11-27	5′-(6FAM)CAACAAGTGGCTTATTT-3′<br>3′- GTTGTTCACCGAATAAA-5′
LE11-27mut	5′-(6FAM)CAACAAGTGTTTTATTT-3′<br>3′- GTTGTTCACAAAATAAA-5′
LE11-27mutC	5′-(6FAM)CAACAAGCGTTTTATTT-3′<br>3′- GTTGTTCGCAAAATAAA-5′
LE11-27mut-5’GC	5′-(6FAM)CGGCAAGTGTTTTATTT-3′<br>3′- GCCGTTCACAAAATAAA-5′
LE11-27mut-5’3’GC	5′-(6FAM)CGGCAAGTGGCGCATTT-3′<br>3′- GCCGTTCACCGCGTAAA-5′
RE11-27	5′-(6FAM)CAACAAGCCACAGGCAA-3′<br>3′- GTTGTTCGGTGTCCGTT-5′
RE11-27T	5′-(6FAM)CAACAAGTCACAGGCAA-3′<br>3′- GTTGTTCAGTGTCCGTT-5′
LE-TIR+13	5′-*AGAGAACAACAACAAGTGGCTTATTTTGA-3′<br>3′-GTCTCTTGTTGTTGTTCACCGAATAAAACT-5′
LE-TIR+30	5′-*AGAGAACAACAACAAGTGGCTTATTTTGATACTTATGCGCCACTTG-3′<br>3′-GTCTCTTGTTGTTGTTCACCGAATAAAACTATGAATACGCGGTGAAC-5′
LE-TIR	5′-*AGAGAACAACAACAAG-3′<br>3′-GTCTCTTGTTGTTGTTC-5′
8+LE-TIR+7	5′-GCGTGAA**AGAGAACAACAACAAGTGGCTTA-3′<br>3′-CGCACTTGGTCTCTTGTTGTTGTTCACCGAAT-5′
8+LE-TIR+30	5′-GCGTGAA**AGAGAACAACAACAAGTGGCTTATTTTGATACTTATGCGCCACTTG-3′<br>3′-CGCACTTGGTCTCTTGTTGTTGTTCACCGAATAAAACTATGAATACGCGGTGAAC-5′
8+RE-TIR+30	5′-GCGTGAA**AGAGAACTTCAACAAGCCACAGGCAAACGTAAGCCACATAGATAAG-3′<br>3′-CGCACTTGGTCTCTTGAAGTTGTTCGGTGTCCGTTTGCATTCGGTGTATCTATTC-5′
8+LE-TIR	5′-GCGTGAA**AGAGAACAACAACAAG-3′<br>3′-CGCACTTGGTCTCTTGTTGTTGTTC(6FAM)-5′
8+LE-TIR+13	5′-GCGTGAA**AGAGAACAACAACAAGTGGCTTATTTTGA-3′<br>3′-CGCACTTGGTCTCTTGTTGTTGTTCACCGAATAAAACT(6FAM)-5′
The star (*) on the top strands indicates a nucleotide gap.<br>The DNAs for EMSA experiments had a 5’-6FAM fluorophore label.	

Supplementary Table 3. Cryo-EM data collection and refinement statistics.

	LE- LE transpososome	RE-RE transpososome
Data collection		
Magnification	130000	106000
Voltage (kV)	200	300
Electron exposure ($e^-/Å^2$)	22.3	48.7
Defocus range (µm)	1 - 2.5	1 - 2.5
Pixel size (Å)	1.16	0.86
Imposed symmetry	none	none
Initial particle number	~3.85 million	~2.92 million
Final particle number	~238300	~53700
Map resolution (Å)<br>FSC threshold	4.6<br>0.143	5.1<br>0.143
Map local resolution range (Å)	4.4 – 7.0	4.7 – 8.5
EMD-ID	28034	40553
Refinement		
Initial model used	8EB5, 6DX0	6DX0, 4D1Q
Model resolution (Å)<br>FSC threshold	4.8<br>0.5	6.9<br>0.5
Model composition non-hydrogen atoms		
- macromolecule	2564	2124
- ligand	6 (Zn)	0
Average B factor ($Å^2$)		
- protein	113.53	264.70
- DNA	128.99	258.47
- ligand	257.16	-
RMSD bond length (Å)	0.003	0.004
Bond angles (°)	0.607	0.659
Molprobity score	1.93	2.18
Clashscore	12.31	18.73
Rotamer outliers (%)	0.05	0.06
Cβ outliers (%)	0	0
Ramachandran plot (%)		
- favored	95	94
- allowed	5	6
- outliers	0	0
CaBLAM outliers (%)	2.86	2.45
PDB ID	8EDG	8SJD
RMSD stands for root mean square deviation.		

Supplementary Table 4. Sequence of the *Hermes* ends of the donor plasmids used in the transposition assay.

Name of the transposon end	Sequence 5' → 3'
LE (wt)	CAGAGAACAACAACAAGTGGCTTATTTTGATACTTATGCGCCACTTGCTA(50)CTTATGAGTACAATTGTGCTTTGCCACTTGA(81)ACAAAAAATTCATTGATTCATCGACACTCGGGTATG(117)TTTTGTCGTGTCGTTCTGCGCAC(140)TCAGTTAAATTTTTTGTCTTACTCTCTTGCTCTCAGCACATCAAGTGTTG(190)TTACTTGTTGTTACTCAGTCGCCTGCCTTATGCTTTTTGGAGA(223)GCGAAAGCACAACGAT(249)CAGAACGGAGAAGTAACAACTTGTTTTGCTAAC(282)AAGTGGCTTATGCACTTGAGTGTG(306)TTTTACACATGTTTTTGAGTTTCGCAGCAAAATGTTCCGATTTGAGCACAATAATTTTACCGTTATTTTGAGTTTTTTAGTTTTGAATAATAAATGTGATTTACTGTTCATCCTCAAAAGAGTTTAAGCAGTAGTAGAGATTAG
RE (wt)	CAGAGAACTTCAACAAGCCACAGGCAAACGTAAGCCACATAGATAAGCACA(50)AGTGTTTTGGGTGTCAAGCCTTTTGAGTGCAAGTATTTTTTTATACACGAGTATTTTTTCACAACTTAACAACAACAGTTGTTTGATGTTAAG(144)ATCACCGCTAGAGTATGAGAGAGTAAAAAGTGTACAAACTCACAAGTGGACGTGTGCGATTTGTCAATTGG(215)CAAATTATACTCACTTCTTGTTGTGTTGTATACTCATTGAACATGAGGGTTGTGTGTGTGTGCTATTGTTTGTAGTATTGCATATGATTCTGTACAAGACTAGGAAAAGAGC(327)ATAAGTATATTAAAAAAAAAAGAAAAATGCTTCTTTTAAACTTAAATTTTTCCAGTCCAAAATTTATTTATTTTTTTTTTTTATTTTAACAAACAAACTTATAAAAAATATTCTTTTTATTTAAAAATGTGA
LEmut	CAGAGAACAACAACAAG**CG**T**AGG**ATTTTGATA**AGC**ATGCGCCACTTGCTACTTATGAGTACAATTGTGCTTTGCCACTTGAACAAAAAATTCATTGATTCATCGACACTCGGGTATGTTTTGTCGTGTCGTTCTGCGCACTCAGTTAAATTTTTTGTCTTACTCTCTTGCTCTCAGCACATCAAGTGTTGTTACTTGTTGTTACTCAGTCGCCTGCCTTATGCTTTTTGGAGAGCGAAAGCACAACGATCAGAACGGAGAAGTAACAACTTGTTTTGCTAACAAGTGGCTTATGCACTTGAGTGTGTTTTACACATGTTTTTGAGTTTCGCAGCAAAATGTTCCGATTTGAGCACAATAATTTTACCGTTATTTTGAGTTTTTTAGTTTTGAATAATAAATGTGATTTACTGTTCATCCTCAAAAGAGTTTAAGCAGTAGTAGAGATTAG
LEΔ	CAGAGAACAACAACAAG(17)C(51)TTATGAGTACAATTGTGCTTTGCCACTTGAACAAAAAATTCATTGATTCATCGACACTCGGGTATGTTTTGTCGTGTCGTTCTGCGCACTCAGTTAAATTTTTTGTCTTACTCTCTTGCTCTCAGCACATCAAGTGTTGTTACTTGTTGTTACTCAGTCGCCTGCCTTATGCTTTTTGGAGAGCGAAAGCACAACGATCAGAACGGAGAAGTAACAACTTGTTTTGCTAACAAGTGGCTTATGCACTTGAGTGTGTTTTACACATGTTTTTGAGTTTCGCAGCAAAATGTTCCGATTTGAGCACAATAATTTTACCGTTATTTTGAGTTTTTTAGTTTTGAATAATAAATGTGATTTACTGTTCATCCTCAAAAGAGTTTAAGCAGTAGTAGAGATTAG
LE50Scr	CAGAGAACAACAACAAGTGGCTTATTTTGATACTTATGCGCCACTTGCTA(50)CTGTCTACATAAAGGCCCCATATTTCCATTATTTCTTAAAAATTAAGTCTCGGGCAGACACCAATATGTAAGCCCTCCATATGATTGGTTTTAAAACGGTCGTTCCACTATTGTGGTGCCTTTAAGTTTTTAAGGGGTCATAGGGTTGATGTTAGATACTTGCATAAGGTCTCTTTGGGGCCTAACCGCTAATGCATTTGACGATGGATATTCTATATGAGATCTCTTTCGTGGTGTAACTGCGTCCTTTTTTTAATGCTATTATTTGTTAAGGAAGGACTAGGTAGATCTGTACGGGTACATCTTACTGTATGTTAGATTTCTGGATCGTCTCCACTCAAGTCTATTACAGTTTACAAATAAGGCTACTGATATTTATGTACTTTCAAATTATGATAT
LE140Scr	CAGAGAACAACAACAAGTGGCTTATTTTGATACTTATGCGCCACTTGCTACTTATGAGTACAATTGTGCTTTGCCACTTGAACAAAAAATTCATTGATTCATCGACACTCGGGTATGTTTTGTCGTGTCGTTCTGCGCAC(140)ATAATTAACTTATCGTGGTTAGTTATGCGTGTGACAAATCTGACGTTTTTCAAAATTGGGTGTTTCTTGGAACATTTTTCACTAAAAAGATAATCTTGGACGTAAGTTTACTAATGAAATCGTGACTGCTGTCTTGATGTTTTTTCTAAATTTTAGTGACGAGCGCCAGGATCGCGGTGTATTGTGTTGCACGATAGTAATGTTCTAACTTACTAACTTTATCATGGGTTGTACCAACTAGTCCTAACTGTTATATGTCGACCTAACTCCACTTTTGAGTTTAACTGTTAATACCTAATTATGTGGCACG
LEmut2	CAGAGAACAACAACAAGTGGCTTATTTTGATACTTATGCGCCACTTGCTACTTATGAGTACAATTGTGCTTTGCC**CAGG**GAACAAAAAATTCATTGATTCATCGACACTCGGGTATGTTTTGTCGTGTCGTTCTGCGCACTCAGTTAAATTTTTTGTCTTACTCTCTTGCTCTCAGCACATC**CCTG**GTTGTT**CAGG**GTTGTTACTCAGTCGCCTGCCTTATGCTTTTTGGAGAGCGAAAGCACAACGATCAGAACGGAG**CCTG**AACA**CAGG**GTTTTGCTAAC**CCTGG**TAGGATGC**CAGG**GAGTGTGTTTTACACATGTTTTTGAGTTTCGCAGCAAAATGTTCCGATTTGAGCACAATAATTTTACCGTTATTTTGAGTTTTTTAGTTTTGAATAATAAATGTGATTTACTGTTCATCCTCAAAAGAGTTTAAGCAGTAGTAGAGATTAG
The mutations in LEmut are in bold. Base pairs at the boundary of sequence deletion or scrambling are numbered.	
